# Molecular and cell-based therapies for muscle degenerations: a road under construction

**DOI:** 10.3389/fphys.2014.00119

**Published:** 2014-04-08

**Authors:** Emanuele Berardi, Daniela Annibali, Marco Cassano, Stefania Crippa, Maurilio Sampaolesi

**Affiliations:** ^1^Translational Cardiomyology Laboratory, Department of Development and Reproduction, KUL University of LeuvenLeuven, Belgium; ^2^Interuniversity Institute of MyologyItaly; ^3^Laboratory of Cell Metabolism and Proliferation, Vesalius Research Center, Vlaamse Institute voor BiotechnologieLeuven, Belgium; ^4^School of Life Sciences, Ecole Polytechnique Fédérale de LausanneLausanne, Switzerland; ^5^Department of Medicine, University of Lausanne Medical SchoolLausanne, Switzerland; ^6^Division of Human Anatomy, Department of Public Health, Experimental and Forensic Medicine, University of PaviaPavia, Italy

**Keywords:** muscle degeneration, molecular treatments, stem cells, gene and cell therapies, cachexia

## Abstract

Despite the advances achieved in understanding the molecular biology of muscle cells in the past decades, there is still need for effective treatments of muscular degeneration caused by muscular dystrophies and for counteracting the muscle wasting caused by cachexia or sarcopenia. The corticosteroid medications currently in use for dystrophic patients merely help to control the inflammatory state and only slightly delay the progression of the disease. Unfortunately, walkers and wheel chairs are the only options for such patients to maintain independence and walking capabilities until the respiratory muscles become weak and the mechanical ventilation is needed. On the other hand, myostatin inhibition, IL-6 antagonism and synthetic ghrelin administration are examples of promising treatments in cachexia animal models. In both dystrophies and cachectic syndrome the muscular degeneration is extremely relevant and the translational therapeutic attempts to find a possible cure are well defined. In particular, molecular-based therapies are common options to be explored in order to exploit beneficial treatments for cachexia, while gene/cell therapies are mostly used in the attempt to induce a substantial improvement of the dystrophic muscular phenotype. This review focuses on the description of the use of molecular administrations and gene/stem cell therapy to treat muscular degenerations. It reviews previous trials using cell delivery protocols in mice and patients starting with the use of donor myoblasts, outlining the likely causes for their poor results and briefly focusing on satellite cell studies that raise new hope. Then it proceeds to describe recently identified stem/progenitor cells, including pluripotent stem cells and in relationship to their ability to home within a dystrophic muscle and to differentiate into skeletal muscle cells. Different known features of various stem cells are compared in this perspective, and the few available examples of their use in animal models of muscular degeneration are reported. Since non coding RNAs, including microRNAs (miRNAs), are emerging as prominent players in the regulation of stem cell fates we also provides an outline of the role of microRNAs in the control of myogenic commitment. Finally, based on our current knowledge and the rapid advance in stem cell biology, a prediction of clinical translation for cell therapy protocols combined with molecular treatments is discussed.

## Introduction

Muscular dystrophies are heterogeneous genetic diseases caused by progressive degeneration of skeletal muscle fibers (Emery, 2002). Mutations in genes encoding for crucial skeletal muscle proteins located either at the plasma membrane (i.e., dystrophin-glycoprotein complex) or, less frequently, within internal cellular membranes are responsible for those disorders. The lack of those proteins increases the probability of damage during contraction and eventually leads to fiber degeneration (Blake et al., 2002; Gumerson and Michele, 2011). Despite the extensive literature reported on this topic, the molecular mechanisms responsible for the progressive muscular degeneration are not yet understood in detail. Physiologically, muscular fiber degeneration is counterbalanced by the regeneration of new fibers formed at the expense of resident myogenic cells and usually each degeneration process is followed by a new regenerative cycle. Skeletal muscle regeneration is mainly sustained by satellite cells (Mauro, 1961), local myogenic progenitors localized underneath the basal lamina of muscle fibers (Tedesco et al., 2010).

When it is damaged, a muscle undergoes a remodeling process and the resident myogenic cells differentiate into myofibroblasts to produce extracellular matrix (ECM), which is required for the adequate tissue repair. Following repeated cycles of degeneration/regeneration, such myofibroblasts accumulate in the muscle producing large amounts of ECM proteins and thus ultimately leading to fibrosis. However, after repeated injuries, the satellite cells in the muscles become exhausted, losing their regenerative capacity. In this view, the genetic manipulation of satellite cells could potentially guarantee an improved muscle regeneration and function. In this review we provide information about the different sources of myogenic stem cells, highlighting their common features and characteristics as well as their controversies in the therapeutic approaches. Advantage and disadvantage for autologous and heterologous cell therapy will be discussed, considering the different sources of myogenic stem cells.

Alterations in skeletal muscle homeostasis can result in either atrophy or hypo-metabolism. Etiologically, the molecular determinants responsible for such metabolic changes are known as common players in different muscular wasting diseases. In this view, they represent promising therapeutic targets common to the wide range of the known muscular diseases that could determine a strong impact in terms of prognosis, clinical setting and management. Pharmacotherapy still represents the most common strategy adopted to counteract muscle wasting for a large spectrum of muscular diseases such as cancer mediated cachexia, Rheumatoid Arthritis (RA) and sarcopenia, while muscular dystrophies can also be potentially treated by a multi-therapeutic approach based on gene/cell therapies combined with molecular treatments.

## Pathophysiology and clinical relevance of muscle wasting

The state of progressive loss of muscular and fat mass known as cachexia syndrome is a condition associated with several chronic diseases such as AIDS, cancer, chronic obstructive lung disease, multiple sclerosis, congestive heart failure, sepsis, diabetes, RA and tuberculosis (Laviano et al., 2003; Fearon et al., 2011). According to its multifactorial and complex nature, as well as to both the pathophysiologic and epidemiologic features of its primary-related disease, such syndrome depicts a worse global epidemiologic scenario if compared with the other musculoskeletal disorders. The dramatic effects that cachexia have on the prognosis are well-known in clinical management of cancer diseases. According with the Global Burden Diseases (GBD) estimations, up to 50% of the oncologic patients suffer from cachexia and up to 80% of them show clear signs of cachexia in the late stages of cancer progression (Laviano et al., 2003; Fearon et al., 2011; Suzuki et al., 2013). Moreover, cancer-related cachexia counteracts the efficacy of radio- and chemotherapeutic treatments by increasing their side effects and decreasing patient's quality of life (Tisdale, 2002). Such complications are directly reliable for a high percentage of mortality in cancer patients, about 20–40%, accounting for more than 2 million of global premature deaths for year (Bruera, 1997).

Among the wide range of the muscular diseases that affect musculoskeletal system by hampering respiratory and locomotive functions, RA, dystrophies and cachexia syndrome represent the most common and are considered as a serious problem for human health. In 2010 GBD estimates showed that musculoskeletal disorders accounted for more than 150,000 deaths, with an increment of 121% between 1990 and 2010 (Lim et al., 2012).

**Figure 1:**
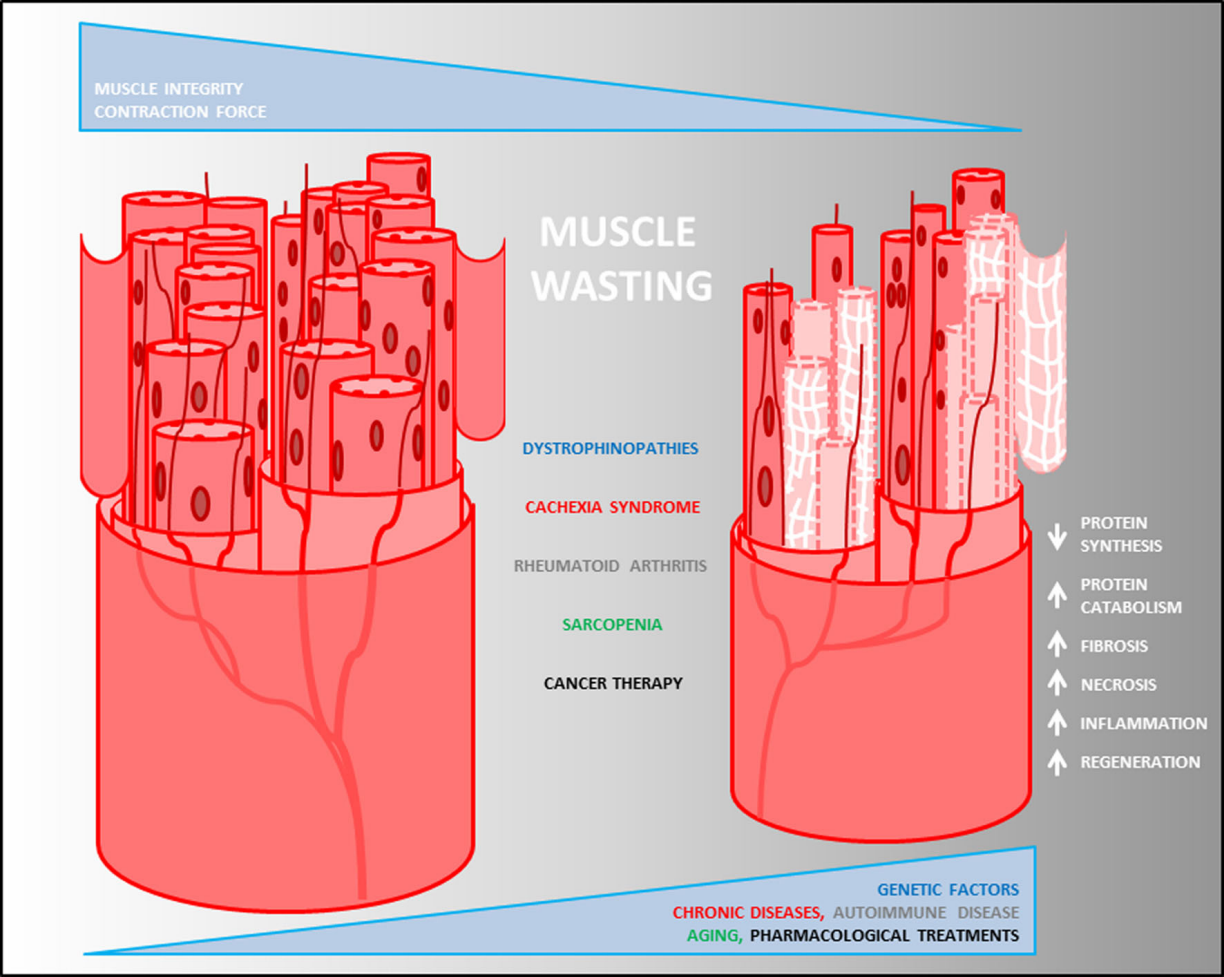
**Pathological heterogeneity of muscle wasting**. Descriptive model of muscle degeneration in chronic diseases. Loss of muscle mass, decrease of fiber size and myonuclear content, reduction of contraction force and increase of fibrosis (white net) are common pathophysiological features of muscle degeneration mediated by changes into the biological process (white arrows) triggered by muscle diseases.

The progressive skeletal muscle weakness and wasting are the main prognostic features exhibited by the heterogeneous musculoskeletal disorders (Leung and Wagner, 2013) (Figure 1). Although musculoskeletal diseases and cachexia have different origins (due to genetic alterations the first and to complications of several chronic diseases the latter), body weight loss, muscle atrophy, fatigue, weakness and loss of appetite are common clinical features observed in both. Nevertheless, while many autoimmune diseases ultimately result in a cachectic state of the patients, they are often associated with unintentional weight loss. RA is an autoimmune disease where the energetic balance is normal and eventually fat mass is increased. Thus, RA is a unique example of autoimmune disease in which cachexia is not associated with a general body-wide wasting and depends exclusively on the reduction in the muscle mass that might be responsible in lowering the average survival of the patients. Therefore, muscle wasting is the key player responsible for the induction of muscle atrophy in musculoskeletal disorders, which is triggered by catabolic events occurring into the affected skeletal muscle tissue (Figure 1). At the molecular level, this is due to an unbalance between protein anabolism and catabolism in favor of proteolysis of some crucial proteins occurring into the muscle fibers, mediated by the expression of muscle-specific ubiquitin ligase (E3 protein) atrogin1/MAFbx and MuRF1 (Bodine et al., 2001; Gomes et al., 2001).

**Figure 2:**
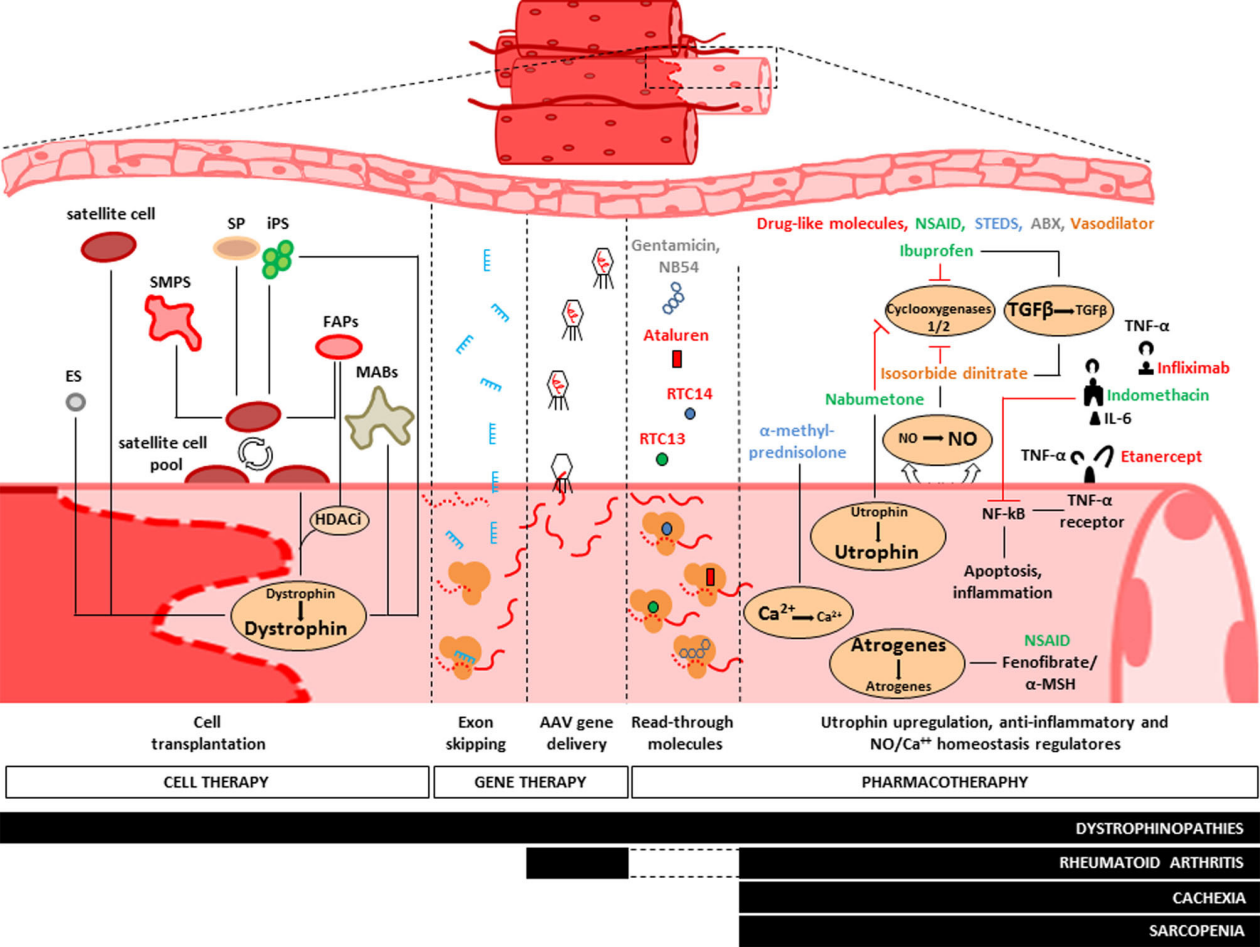
**Treatments of muscle diseases**. Representative scheme of the main therapeutic approaches adopted to counteract muscle wasting. Pharmacotherapy aims to maintain muscle integrity by neutralization of ubiquitin-proteasome pathway (UPP), provoked by circulating pro-inflammatory stimuli (i.e., TNF-α and Il-6). Administration of non-steroidal anti-inflammatory drugs (NSAID, green), fenofibrates and steroids (STEDS, blue) reduces the overall expressions of atrogenes. It also stimulates utrophin expression and regulates the cytosolic homeostasis of NO and Ca^++^ elements, while drug-like molecules (red) and antibiotics (green) provide the “read-through” strategy to obtain semi-functional dystrophin protein. To date, NSAID and STEDS are the most diffused drugs to treat dystrophinopathies, cachexia syndrome, rheumatoid arthritis and sarcopenia. Gene therapy is experimentally adopted for dystrophinopathies treatments. Such method is based on the use of Adeno-associated viruses (AAV) and lentiviral vectors to mediate the delivery of micro-dystrophin or mini-utrophin and by use of exon skipping strategy to increase the endogenous expression of dystrophin (see text). Skeletal Myogenic Precursors (SMPS), Side Population (SP), Fibro-Adipogenic Progenitors (FAPs) and Mesoangioblasts (MABs) are potential candidates for cell therapeutic approaches of dystrophinopathies. MABs were recently enrolled in PhaseI/II clinical trial either for their ability to repopulate the endogenous pool of satellite cells and for their myogenic differentiation capability to produce dystrophin.

Beyond the basic action of mechanic contraction, the skeletal muscle is a tissue involved in many other metabolic activities such as glucose, glycogen and lipid metabolism (Jensen and Richter, 2012), as well as endocrine (Pedersen and Febbraio, 2008) and immunogenic activities (Nielsen and Pedersen, 2008). Such biological heterogeneity reflects the histological diversity observed into the skeletal muscle tissue and, in turn, highlights multifaceted possibilities for the therapeutic interventions. It has been recently demonstrated that the microenvironment outside the myofibers can actively participate to the cancer-mediated muscle wasting. This happens when circulating tumor factors induce muscle damage by activation of both satellite and non-satellite muscle progenitor cells, and such process is followed by inhibition of their myogenic differentiation due to a persistent expression of Pax7 (He et al., 2013). On the other hand, the metabolic complexity of the skeletal muscle also renders it susceptible to environmental stimuli. Epidemiological studies show indeed the potential role of environmental and lifestyle factors (i.e., physical activity, diet and sun exposure) on the increasing susceptibility of the insurgence of sarcopenia (Scott et al., 2011). Overall, studies focused on the investigation of the general molecular mechanisms responsible for muscle wasting identified some potential therapeutic targets involved in the main catabolic pathways and that could be inhibited by pharmacological and by gene- or cell-therapy based approaches. Specifically, we will discuss pharmacological strategies aimed to counteract the effects of pro-inflammatory stimuli (i.e., TNF-α, IL-6) in cachexia, sarcopenia and RA, as well vector-based micro-dystrophin transfer, oligonucleotide-induced exon-skipping and cell therapy strategy based on the use of healthy myogenic cell precursors [i.e., satellite cells, side population (SP), fibro-adipogenic progenitors (FAPs), mesoangioblasts, ES, and iPS cells] in dystrophinopathies (Figure 2).

## Pharmacological approach

Lack or alteration of structural proteins into the musculoskeletal system causes chronic inflammation. Although there are no specific cures for muscle wasting mediated by the different forms of muscular dystrophies and cachexia, pharmacotherapy has been the first historical clinical approach used to modulate the progression of such diseases (Abdel-Hamid and Clemens, 2012) by counteracting chronic inflammation (Figure 2). Because dystrophin plays a crucial role in preserving the integrity of the muscular membrane by permitting the anchorage of the dystrophin-associated protein complex, lack or genetic mutations of dystrophin result in a chronic influx of calcium into the myofibers, causing cellular death and inflammatory responses. In addition, fibrosis can occur to replace the damaged muscular fibers, causing muscle weakness (Figure 1). Pilot studies performed in patients affected by Duchenne/Becker and Limb-Girdle muscular dystrophies (DMD, BMD, and LGMD respectively) based on the administration of non-steroidal anti-inflammatory drugs, such as ibuprofen and nabumetone, or on the use of isosorbide dinitrate, a NO donor vasodilator, showed an improvement of the general pathophysiologic conditions (Figure 2). This effect was mediated by a deregulation of circulating level of TGF-β (D'Angelo et al., 2012), a known mediator of fibrosis in dystrophinopathies (Goldstein and Mcnally, 2010). Corticosteroids have been proposed as a pharmacological therapy for dystrophinopathies, in order to counteract muscle necrosis, inflammation and to reduce the muscle membrane susceptibility to damage (Abdel-Hamid and Clemens, 2012). In particular, prednisone (Griggs et al., 1991, 1993; Bonifati et al., 2000) and deflazacort (Bonifati et al., 2000) induce improvement and a long-term stabilization of the muscle strength (Bonifati et al., 2000), as well as a substantial reduction of weakness progression in DMD patients (Moxley et al., 2005). Moreover, since the elevation of cytosolic calcium concentration can trigger apoptotic and/or necrosis events in the dystrophic muscles, such physiological alteration represents another important glucocorticoid-based therapeutic target. Recently, studies in preclinical models proposed α-methylprednisolone, administrated either alone (Ruegg et al., 2002) or in combination with taurine (an aminoacid with antioxidant properties), as a candidate for pharmacological regulation of the cytosolic calcium flux in dystrophic muscles (Cozzoli et al., 2011).

The pharmacological efforts aiming to counteract muscle degeneration in dystrophinopathies mainly point to stabilize the muscular membrane integrity. This is the case of drugs designed to increase the expression level of native utrophin, as a mechanisms used to compensate for the dystrophin lack (Tinsley et al., 1998; Gilbert et al., 1999). Nabumetone is a novel promising small molecule with anti-inflammatory properties (COX1 and 2 inhibitor) that *in vitro* can activate the promoter of the A isoform of utrophin (Moorwood et al., 2011). The administration of aminoglycosides antibiotics (i.e., Gentamicin, NB54) (Barton-Davis et al., 1999; Politano et al., 2003; Nudelman et al., 2009) and read-through compounds such as RTC13, RTC14 (Kayali et al., 2012), or ataluren (PTC124) (Hamed, 2006; Finkel, 2010) has been proposed as a new strategy to induce ribosomal read-through of premature termination mutations, to obtain a full-length dystrophin protein in patients with DMD and Becker Muscular Dystrophy (BMD) (Figure 2). Various pro-inflammatory stimuli are involved in cancer mediated muscle wasting (Todorov et al., 1996; Suzuki et al., 2013), RA (Gomez-Sanmiguel et al., 2013) and sarcopenia (Malafarina et al., 2012). In this case the pharmacological approaches used so far aim to counteract the biological activity of secreted pro-inflammatory mediators, such as interleukins (Il-1β, IL-6), interferon gamma (IFN-γ), tumor necrosis factor alpha (TNF-α) (Todorov et al., 1996) and proteolysis inducing factor (PIF) (Todorov et al., 1999). Unfortunately, anti-cytokine therapy aimed to block TNF-α by administration of Infliximab (monoclonal TNF antibody) or Etanercept (soluble TNF-α receptor) in cancer patients showed only poor ameliorative effects on cachexia pathophysiology (Gueta et al., 2010; Wu et al., 2013), whereas in patients with RA mediated cachexia, Etanercept was shown to reduced mortality (Morgan et al., 2014) and ameliorate the muscular function (Marcora et al., 2006). Indomethacin showed anti-cachectic effects in muscles from tumor bearing mice by inducing reduction in the levels of NF-kappaB, TNF-α and IL-6 (Zhou et al., 2003). Notably, dithiocarbamate inhibits IL-6 synthesis (Nai et al., 2007). Other treatments proposed in *in vivo* models in order to counteract oxidative and inflammatory burden in cancer-mediated muscle wasting are based on administration of glycine (Ham et al., 2013), simvastatin (Palus et al., 2013), eicosapentaenoic acid (Vaughan et al., 2012) and use of proteasome inhibitors to block the ubiquitin-proteasome pathway (Zhang et al., 2013). Such treatments efficiently counteract the expression of genes associated with the muscle protein breakdown observed in cancer cachexia (i.e., Atrogin-1 and MuRF-1) On the contrary, fenofibrate, a PPARα agonist (Castillero et al., 2011), and α-Melanocyte-stimulating hormone (α-MSH) (Gomez-Sanmiguel et al., 2013) ameliorate the pathophysiology of muscles in an adjuvant-induced arthritis rat model by preventing the overexpression of Atrogin-1, MuRF-1, and myostatin observed in RA (Castillero et al., 2011; Gomez-Sanmiguel et al., 2013). Pharmacological treatments used to counteract the progressive loss of skeletal muscle mass observed in sarcopenia are based on the administration of ghrelin, testosterone, Growth Hormone (GH), myostatin inhibitors and supplementation of vitamin D (Malafarina et al., 2012). Therapeutically, despite the efforts spent so far for sarcopenia treatment, only few results have been achieved in terms of increased muscle mass and strength, and decrease of muscle catabolism. Because vitamin D levels decrease with elderly, promising results were obtained in dietary supplementation of vitamin D in aged people, specially in muscle functional improvement (Malafarina et al., 2012).

## Gene therapy

Gene replacement strategy was historically conceived to counteract the lack of dystrophin that affects DMD and BDM patients. Transgenic mice (mdx), dogs with X-linked muscular dystrophy (GRMD), and non-human primates (cynomolgus macaques) are examples of animal models extensively used to test novel methods for dystrophin gene delivery. Adeno-associated viruses (AAV) and lentivirus based vectors mediate efficient delivery of micro-dystrophin or mini-utrophin (Cerletti et al., 2003) and provide an alternative option for dystrophin-deficient mdx mouse (Gregorevic et al., 2004, 2006; Yoshimura et al., 2004; Liu et al., 2005; Rodino-Klapac et al., 2007), in non-human primate animal models (Rodino-Klapac et al., 2007) and Golden Retriever Muscular Dystrophy (GRMD) dogs (Cerletti et al., 2003; Sampaolesi et al., 2006; Koo et al., 2011). Nevertheless, all the dystrophin delivery methods proposed so far showed poor restoration of dystrophin within a small area of the skeletal muscle tissue targeted and only a partial improvement of the contractile properties (Rodino-Klapac et al., 2013) (Figure 2). Since deletions of single or multiple exons in the dystrophin gene are the most pathogenic mutations in DMD and BDM, antisense-mediated exon skipping (Douglas and Wood, 2013) represents a promising additional strategy adopted to increase dystrophin expression in DMD and BDM models, by restoring the genetic reading frame. Notably, this can be obtained either by single- (Van Deutekom et al., 2007; Jorgensen et al., 2009; Kinali et al., 2009) or multi-exon skipping approaches (Aartsma-Rus et al., 2006; McClorey et al., 2006; Goyenvalle et al., 2012). So far, many antisense oligonucleotide, such as morpholino oligomers (PMOs) and 2′O-methylphosphorothioate oligoribonucleotides (2′OMe), have been synthetized and successfully tested both *in vitro* and *in vivo* (Benedetti et al., 2013). They act by targeting of specific exons allowing their skipping during the splicing of dystrophin mRNA (Figure 2). In 2007 van Deutekom and colleagues tested the ability of PRO051oligonucleotide to restore dystrophin into the *tibialis anterior* of 4 DMD patients. In 2009 Kinali and colleagues treated the *extensor digitorum brevis* of 7 DMD patients with morpholino splice-switching oligonucleotide (AVI-4658) (Kinali et al., 2009). These trials provided evidences for local restoration of dystrophin in the treated muscles and for the safety of the protocols adopted (Van Deutekom et al., 2007).

However, because myoastin negatively affects skeletal muscle growth, AAV-mediated gene delivery of myostatin inhibitors (i.e., MRPO) has been proposed as a therapeutic strategy to maintain muscle mass (Morine et al., 2010) and improve the contraction force (Qiao et al., 2008) in both mdx mice (Qiao et al., 2008; Morine et al., 2010) and dogs (Qiao et al., 2009). Noteworthy, gene therapy AAV-mediated approaches were also used to restore structural, such as sarcoglycans (Sampaolesi et al., 2003), and non-structural proteins (Goonasekera et al., 2011). In fact, it is known that cytosolic alteration of Ca^2+^ flux observed in muscular dystrophies leads to sarcolemmal instability. This could be reduced by overexpressing the sarcoplasmic reticulum Ca^2+^ ATPase 1 (SERCA1) in both mdx and δ-sarcoglycan-null (*Sgcd^−/−^*) mice (Goonasekera et al., 2011). Preclinical studies about the therapeutic applications of AAV-based gene delivery strategies were also performed to treat RA (Dai and Rabie, 2007). In particular, because the synovial lining is poorly transduced, subsynovial muscle tissues have been predominantly transfected in various RA models to investigate the effects of anti-inflammatory mediators such as IL-4 (Cottard et al., 2000) and IL-10 (Apparailly et al., 2002) either in mice (Cottard et al., 2000; Apparailly et al., 2002) as well as in human and murine synovial cell lines (Katakura et al., 2004).

## Cell therapy

As already previously mentioned, satellite cells are quiescent unipotent stem cells, located underneath the basal lamina of adult skeletal muscle fibers (Mauro, 1961). They are formed during the second wave of embryonic myogenesis after which they exit the cell cycle, contributing significantly to the first post-natal muscle growth (Gros et al., 2005; Kassar-Duchossoy et al., 2005; Relaix et al., 2005). In case of muscle damages, satellite cells can re-enter the cell cycle resulting in an increasing number of myogenic progenitors able to fuse and form new muscle fibers (Huard et al., 2002; Jarvinen et al., 2005; Tedesco et al., 2010; Wang and Rudnicki, 2012). Given their natural commitment, it has been easy to consider satellite cells as the leading candidate for muscle regeneration in dystrophic mice (Partridge et al., 1989). In the case of transplantation of individual fibers into the *tibialis anterior* of irradiated mdx mice (specific treatment used to remove the existing population of satellite cells), satellite cells of the fiber donor expand, repopulating the endogenous pool, and differentiate into functional myofibers. Pax7^+^/CD34^+^/GFP^+^ satellite cells, isolated from the diaphragm of Pax3::GFP mice, proved a good cellular model for the treatment of irradiated mdx muscles, resulting in restoration of the expression of dystrophin in many skeletal fibers and reconstitution of the pool of resident satellites cells (Montarras et al., 2005) (Figure 2). It has been shown by *in vivo* imaging that a single CD34^+^/integrinα^+^ satellite cell can replenish the resident satellite pool that, in the presence of further damage, can quickly re-enter a new wave of proliferation, generating new myofibers (Sacco et al., 2008). Fifty years of studies about the role of stem cells in skeletal muscle regeneration have identified a complex pattern of expression for surface markers within the endogenous pool of satellite cells (Rinaldi and Perlingeiro, 2013). Specific subpopulations of satellite cells positive for CD34 (Beauchamp et al., 2000), M-cadherin (Irintchev et al., 1994), α7β 1-integrin (Burkin and Kaufman, 1999; Gnocchi et al., 2009), syndecan-3/4 (Cornelison et al., 2004), the chemokine receptor CXCR4 (Ratajczak et al., 2003) barx2 (Meech et al., 2012) and caveolin-1 (Volonte et al., 2005) have been identified and investigated for their *in vitro*, as well as *in vivo*, therapeutic potential.

The intra-muscular injections, however, have revealed some significant problems. One of the major limitations on the use of satellite cells in therapy is the cell heterogeneity. The variable rate of satellite cell homing after transplantation of a single myofiber could be due to the cell heterogeneity and their functional niche of origin. Moreover, satellite cells show limited migratory ability, reduced myogenic capacity when expanded *in vitro*, while the host immune rejection still represents a major issue when satellite cells are used for transplantation (Mouly et al., 2005; Kuang and Rudnicki, 2008). Nevertheless, many others methodological issues concerning intramuscular cell transplantation of myogenic stem cells in the treatment of dystrophinopathies have been partially solved by a further basic knowledge of the skeletal muscle stem cell biology, achieved in the recent years by both clinical and pre-clinical data (Hill et al., 2006; Skuk and Tremblay, 2011; Brack and Rando, 2012).

Recently, interesting results have been obtained by the prospective isolation of skeletal myogenic precursors (SMPS) (Figure 2), a distinct population of the satellite cells pool, characterized by the expression of Cxcr4 and β 1integrin and the absence of CD45, Sca1 and Mac1(Sherwood et al., 2004). Once injected into immunodeficient mdx muscles, SMPS contribute to the muscle regeneration (up to 94%) and to the satellite cell pool. SMPS need to be injected freshly isolated, in order to have beneficial effects. Although their migratory capacity remains limited to the areas surrounding the site of injection, the contractile force of limb muscles was significantly higher in the treated mice compared to controls (Cerletti et al., 2008).

A subpopulation of progenitors associated with skeletal muscles is a so-called SP. SP cells are defined as myogenic cells SCA1^+^/ CD45^+^ (Polesskaya et al., 2003; Seale et al., 2004), unable to retain the intercalating Hoechst 33342 dyes (Gussoni et al., 1999; Polesskaya et al., 2003; Montanaro et al., 2004). When co-cultured with myoblasts, they can fuse to form myotubes *in vitro* and, if injected into the femoral artery in mice mdx5^cv^ mice, can contribute up to 5–8% of the regenerated fibers (Perez et al., 2009). Remarkable results were obtained with the isolation of a rare subset (0.25%) of SP cells, identified as SCA1^+^/ABCG2^+^/Syndecan4^+^/Pax7^+^ cells (Tanaka et al., 2009). Once injected, those cells in muscle treated with 1.2% BaCl_2_ regenerate up to 30% of the fibers and, surprisingly, reconstitute up to 75% of the endogenous satellite pool (Tanaka et al., 2009). However, the muscle damage induced by BaCl_2_ is not a commonly accepted model of regeneration and this must be taken into account when interpreting these results. Another class of myogenic precursor was isolated from the population of endothelial cells in human muscles, through the prospective isolation of cells by FACS CD56^+^/CD34^+^/CD144^+^ (Okada et al., 2008). These myoendothelial progenitors, after injection into injured muscle of SCID mice can be grafted into existing muscle fibers and form neofibres (Tamaki et al., 2002). A population of muscle-interstitial cells, referred to as FAPs, was recently identified. FAPs have been shown to mediate the beneficial effects of histone deacetylase inhibitors (HDACi) in mdx mice (Mozzetta et al., 2013). HDACi are known as promoters of endogenous regeneration and functional recovery of dystrophic muscles in the mdx mouse. They act by increasing the fiber size and reducing both the fibrosis and the fat deposition (Minetti et al., 2006). FAPs isolated from young dystrophic subjects show a minimal myogenic commitment that can be implemented by HDACi at the expense of their fibro-adipogenic potential. In addition, HDACi enhance FAPs ability to promote differentiation of adjacent satellite cells (Figure 2).

In recent years, a new class of stem cells associated with vasculatures and termed mesoangioblasts (MABs) have been studied as potential therapeutic protocols to threat dystrophic muscles (Figure 2). MABs were originally isolated from the dorsal aorta of the embryo (E9.5) (Minasi et al., 2002) and then from adult skeletal muscles in mice, dogs, and humans (Tonlorenzi et al., 2007; Quattrocelli et al., 2012). MABs are positive for several markers, including CD34, SMA, Pdgfrα, Pdgfrβ, Ng2, and AP, supporting the hypothesis that they belong to a subgroup of pericytes. MABs are multipotent as highlighted by their myogenic, osteogenic, chondrogenic, and adipogenic differentiation potential observed in quail-chick chimeras and *in vitro* and *in vivo* experiments in mouse model. After intra-arterial injection in dystrophic muscles of *Scga-*null mice or GRMD dogs, MABs are able to regenerate (up to 50%) muscle architecture, with functionality (Sampaolesi et al., 2006). Similarly, promised results were obtained with the transplantation of human MABs in immunodeficient mdx mice (Sampaolesi et al., 2003). Building up on those promising preclinical studies a Phase I/II clinical trial of donor mesoangioblasts transplantation from HLA-identical donors in 5 DMD patients is nearing completion (EudraCT Number: 2011-000176-33). However, due the ethical rules, several limitations are present in this first attempt of stem cell systemic delivery in DMD patients. First, the age of the patients and progression of the disease were advanced in the enrolled patients. Second, cell dose was quite low, from 1/5 to 1/10 of that administered to the GRMD dogs. Third, injections were limited into the femoral arteries, confining the cell treatment mainly to the muscles downstream the femoral artery. Taken into account those limitations, this trial will give important information about the safety of systemic delivery of adult stem cells in DMD patients. This trial will also hopefully answer some questions regarding the capability of donor stem cells to migrate towards regenerating muscles and undergo myogenic differentiation, by producing dystrophin.

In the last few years there is a growing interest about the therapeutic prospective offered by pluripotent stem cells. Embryonic Stem (ES) cells have been isolated from the inner cell mass of the blastocyst in mouse (Evans and Kaufman, 1981; Martin, 1981) and in human (Thomson et al., 1998), showing pluripotent features. Mouse and human ES cells can efficiently differentiate into the three germ layers, mesoderm, ectoderm and endoderm and a consistent number of studies showed their myogenic differentiation potential *in vitro* and *in vivo* (Bhagavati and Xu, 2005; Barberi et al., 2007; Filareto et al., 2012). The therapeutic use of ES cells has been strongly debated in the scientific community, mainly because of limitations linked to immune rejection and ethical concerns. However, part of these obstacles has been overcome by a pioneering study of Yamanaka in 2006 (Takahashi and Yamanaka, 2006) showing that induced pluripotent stem (iPS) cells can be generated from somatic cells. In addition, a very recent paper published in Nature showed that is possible to generate pluripotent stem cells by STAP, stimulus-triggered acquisition of pluripotency (Obokata et al., 2014). Basically, pluripotent conversion can be achieved by brief exposition of low-passage source cells to acidic conditions (pH 5.7). Since STAP reprogramming takes a very short period, only few days unlike transgene- or chemical-induced iPS cell conversion, it could be clinically relevant for tailoring cell therapy approaches.

To date, the use of iPS cells to potentially correct the dystrophic phenotype has been reported in several studies on murine (Mizuno et al., 2010; Darabi et al., 2011; Quattrocelli et al., 2011; Filareto et al., 2013) and human iPS cells (Darabi et al., 2012). Preclinical evidences show that myoblasts and mesenchymal cells derived from human ES and iPS can efficiently fuse with mature muscle fibers (Awaya et al., 2012; Goudenege et al., 2012) and improve the performances of engrafted muscles (Darabi et al., 2012) (Figure 2). After introduction of factor-based reprogramming, generation of iPS cells is feasible from any kind of cell population. However, iPS cells own an epigenetic memory, which results in a biased cell-differentiation towards the cell lineage of its source. This is probably due to the conservation of epigenetic marks, like CpG island (CGI) methylation and histone modifications, after reprogramming (Kim et al., 2010; Polo et al., 2010). Also iPS cells generated from murine skeletal MABs upon teratoma analysis differentiated with a significantly greater efficiency towards skeletal myocytes compared to fibroblast-reprogrammed iPS cells (Quattrocelli et al., 2011). If the myogenic memory will be confirmed in human iPS cells, this phenomenon could have an unpredicted impact for future translational studies.

Overall, these evidences suggest that, although all the efforts made so far on the use of pluripotent stem cells have been focalized on dystrophinopathies, the therapeutic use of iPS and ES cells could be an extraordinary potential clinical tool useful in the treatment of any skeletal muscle degenerations.

## New challenge: microRNAs and their therapeutic potential

In the early '90 a 22 nt non-coding transcript RNA, lin-4, was identified in *C. elegans*. It represses the expression of lin-14, a nuclear protein necessary for the larval development (Lee et al., 1993; Wightman et al., 1993). This discovery was the trigger for thousands of subsequent publications regarding the identification of micro inhibitory RNAs (miRNAs) in early embryogenesis (Berardi et al., 2012) and in cardiac and skeletal myogenesis (Ge and Chen, 2011; Crippa et al., 2012). miRNAs target sites in the 3'UTR of the mRNA leading to inhibition of mRNA translation and/or enhanced mRNA degradation, thus resulting in the decrease of protein expression levels (Djuranovic et al., 2011). miRNAs are generally transcribed by RNA polymerase II, as a primary miRNA (pri-miRNA)s then the RNase III enzyme Drosha removes hairpins from pri-miRNAs generating pre-miRNAs. In the cytoplasm, the pre-miRNA is further cleaved by the RNase III enzyme Dicer to generate mature miRNAs.

**Figure 3:**
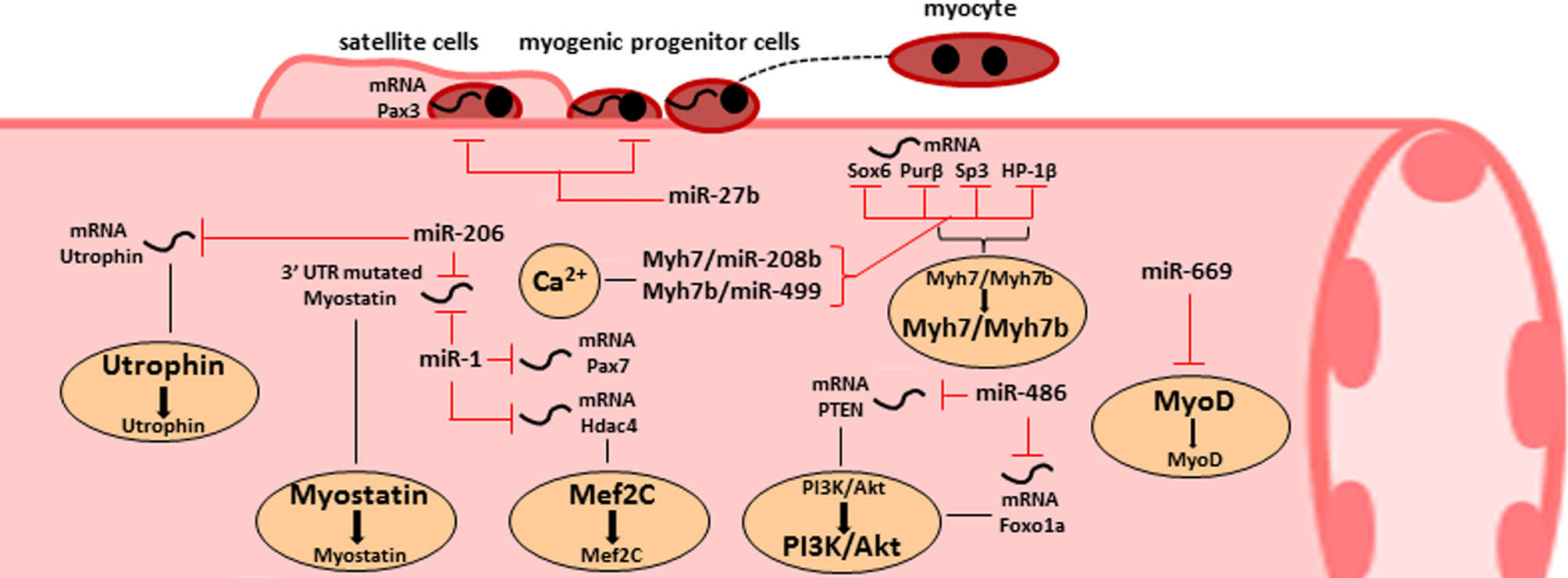
**The roles of miRNA in skeletal muscle homeostasis and dysfunction**. Representative scheme of the main miRNAs involved in skeletal muscle functions. Specific miRNAs are critical for satellite cell activation (miR-27b) or for skeletal muscle differentiation (miR-1) and can be induced for therapeutic approaches. Other miRNAs are involved in muscle metabolism and they are able to modulate the AKT/PI3K pathway. Two miRNA families are peculiar for their opposite dual biological functions: miR-669 can inhibit MyoD causing a benefit in cardiac progenitors but in the same time reduced myogenic potential in skeletal muscle progenitors; miR-206 can induce hypertrophy by targeting mutated 3'UTR of myostatin messengers and can sustain dystrophic phenotype by inhibiting utrophin expression.

Highly expressed miRNAs in skeletal muscle tissue are termed myomiRs, which include miR-1, miR-133a, miR133-b, miR-206, miR-208, miR208b, miR486, and miR-499 (Van Rooij et al., 2008). They can be responsible for metabolic changes in skeletal muscle tissue (Figure 3). For example, muscular hypertrophy in Texel sheep is caused by a point mutation in the 3'UTR of myostatin RNA messengers, which creates a target site for miR-1 and miR-206 (Clop et al., 2006). Those miRNAs are abundant in the skeletal muscle tissue and can negatively affect myostatin expression, one of the most effective repressor of muscle growth. However, since another possible target for miR-206 is utrophin, a valuable substitute of dystrophin, several researchers believe that miR-206 can be responsible to sustain the dystrophic phenotype (Rosenberg et al., 2006). Transgenic mice for gain and loss of function studies are necessary to shed light in those controversies.

miRNA are also critic regulators for muscle wasting. In fact, possible targets for miR-486 are PTEN (phosphatase and tensin homolog) and FoxO1A, elements of the PI3/Akt pathway (Small et al., 2010) involved in muscle wasting and apoptosis. The activity of miR-486 results in muscle hypertrophy, since it leads to the activation of mTOR via PTEN inhibiton, and to the reduction of the ubiquitin ligase expression, which sustains atrophy in skeletal muscle tissue. The expression profiles of miR-486 strongly support its role in muscle homeostasis, since it is decreased in denervated muscles and almost absent in Duchenne patients (Eisenberg et al., 2007).

miR-1 and miR-133 modulate skeletal and cardiac muscle growth and differentiation. miR-1 promotes skeletal muscle differentiation by targeting the histone deacetylase 4 (*HDAC4*), that in turn represses Mef2C, an essential muscle transcription factor. On the contrary, miR-133 stimulates myoblast proliferation by targeting *SRF* (Chen et al., 2006), while miR-206 promotes myoblast differentiation targeting the mRNA of *PolA1* (Kim et al., 2006), a DNA polymerase subunit. MyoD and myogenin can regulate miR-206 expression by binding specific elements in the enhancer region upstream miR-206 gene. Overexpression of miR-27b causes premature differentiation of muscle satellite cells: once myoblasts exit the cell cycle, miR-27 indeed targets the 3'UTR of *Pax3* (Crist et al., 2009).

The fine-tuned expression of miR-499 and miR-208b plays a role in the control of skeletal muscle performance. In response to calcium signaling, miR-208b and miR-499 indeed reinforce slow fiber conversion by inducing the expression of β-MHC and Myh7b (Van Rooij et al., 2009). They were considered initially as MyomiRs since they are encoded by introns of their host myosin genes Myh7 and Myh7b. These two intronic miRNAs target the transcriptional repressors of slow myofiber genes, including Sox6, Purβ, Sp3, and HP-1β, (Van Rooij et al., 2009). Recently, we have also identified a microRNA family, called miR-669, involved in the muscle lineage switch (Crippa et al., 2011) and used them as therapeutic molecules for long term treatments (Quattrocelli et al., 2013) in an animal model of limb girdle muscular dystrophy type 2E. Up to now, crucial information concerning the effects of miR669 in human setting for cardiac and skeletal muscle differentiation are still missing.

In summary, the possibility to increase the pool of myogenic stem cells, induce hypertrophy or reduce atrophic cell signaling by the perturbation of miRNA expression profiles offers a new opportunity to re-establish the skeletal muscle homeostasis lost in the wide range of muscolo-skeletal disorders (Figure 3).

## Concluding remarks

In conclusion, stem cell research will ride the third millennium as highlighted by the Nobel Prize in Physiology or Medicine 2012 jointly conferred to John Gurdon and Shinya Yamanaka for their discoveries on cell reprogramming which paved the way for new therapeutic horizons. We accumulated evidences that stem cell therapy with donor adult cells has produced dramatic amelioration in dystrophic mice and dogs. However, their finite lifespan and replication capacity, limit their therapeutic potential. Several types or subtypes of resident stem cells have been isolated and characterized from adult skeletal muscles. Further translational studies are still necessary to get molecular insights on how to improve the myogenic potential of each cell type. Moreover, it will be relevant to reveal the molecular and epigenetic signatures of myogenic progenitors to identify all molecules involved in the crosstalk among the different pools. In addition, several articles have documented a subset of miRNAs that regulate myogenic cell proliferation, differentiation, and contractility. The possibility to improve myogenic commitment of stem cells by targeting the expression of specific miRNAs is now implicated in several preclinical studies (Crippa et al., 2012). New insights into additional mechanism of post-transcriptional regulation mediated by lncRNAs are desirable since they have an impact on the distribution of miRNA molecules on their targets (Twayana et al., 2013). In the following years miRNA technologies combined to stem cell treatments will test novel therapeutic strategies for skeletal muscle disorders.

Muscle progenitors may be generated from patient iPS cells, genetically modified, systemically injected, then recruited to and integrated in the areas of damage. This would circumvent problems related to allogeneic transplantation and difficulty in obtaining autologous stem cells. The novel reprogramming methods (Obokata et al., 2014) do not require nuclear transfer or genetic manipulation and thus they are more suitable for translational studies with clinical implications. It is interesting that such a great potential has not been explored yet in cachexia and sarcopenia, where it could be employed avoiding genetic manipulation. However, the enormous research impetus on regenerative medicine and stem cell-based therapy could strongly influence the future scientific directions. Emerging literature supports the hypothesis that downregulating myonuclear apoptosis might preserve muscle mass and function in the elderly. In principle, employing pharmacological or genetic interventions to target muscle protein turnover, autophagy and myogenic stem cell function may provide a more thorough protection against muscle aging and atrophy. These multi-therapeutic approaches will face several challenges, including the clear determination of feasible therapeutic windows for each specific intervention, especially if systemic delivery is employed. Nevertheless, pursuing this path is certainly worth in order to relieve the individual and societal burden associated with muscular degeneration.

### Conflict of interest statement

The authors declare that the research was conducted in the absence of any commercial or financial relationships that could be construed as a potential conflict of interest.
